# D-helix influences dimerization of the ATP-binding cassette (ABC) transporter associated with antigen processing 1 (TAP1) nucleotide-binding domain

**DOI:** 10.1371/journal.pone.0178238

**Published:** 2017-05-23

**Authors:** Ahmet S. Vakkasoglu, Sriram Srikant, Rachelle Gaudet

**Affiliations:** Department of Molecular and Cellular Biology, Harvard University, Cambridge, Massachusetts, United States of America; University of Technology Sydney, AUSTRALIA

## Abstract

ATP-binding cassette (ABC) transporters form a large family of transmembrane importers and exporters. Using two nucleotide-binding domains (NBDs), which form a canonical ATP-sandwich dimer at some point within the transport cycle, the transporters harness the energy from ATP binding and hydrolysis to drive substrate transport. However the structural elements that enable and tune the dimerization propensity of the NBDs have not been fully elucidated. Here we compared the biochemical properties of the NBDs of human and rat TAP1, a subunit of the heterodimeric transporter associated with antigen processing (TAP). The isolated human TAP1 NBD was monomeric in solution, in contrast to the previously observed ATP-mediated homodimerization of the isolated rat TAP1 NBD. Using a series of human-rat chimeric constructs, we identified the D-helix, an α-helix N-terminal to the conserved D-loop motif, as an important determinant of NBD dimerization. The ATPase activity of our panel of TAP1 NBD constructs largely correlated with dimerization ability, indicating that the observed dimerization uses the canonical ATP-sandwich interface. The N-terminus of the D-helix from one protomer interacts with the ATP-binding Walker A motif of the second protomer at the ATP-sandwich interface. However, our mutational analysis indicated that residues farther from the interface, within the second and third turn of the D-helix, also influence dimerization. Overall, our data suggest that although the D-helix sequence is not conserved in ABC transporters, its precise positioning within the NBD structure has a critical role in NBD dimerization.

## Introduction

ATP-binding cassette (ABC) proteins form a large and ubiquitous superfamily, with bacterial, archaeal and eukaryotic genomes each encoding a wide variety, including 49 in the human genome [1, 2]. All ABC proteins share a common feature—a similar nucleotide-binding domain (NBD) [3–5]. Most ABC superfamily members are transporters, including both exporters and importers, with a few additional ABC proteins that perform non-transport functions [6]. ABC transporters transport various substrates across the cellular membranes by using ATP to energize the process [3, 7].

The common ABC transporter architecture includes a transmembrane domain (TMD) typically formed of two homo- or heteromeric repeats, and a cytoplasmic energy-generating unit which consists of either homo- or heterodimeric NBDs [5]. The TMDs are generally associated with substrate binding and translocation, whereas the NBDs bind and hydrolyze ATP. Several high-resolution structures confirm this general architecture in both exporters (e.g. [8, 9]) and importers (e.g. [10–13]; see [14] for a recent review). ABC transporter structures revealed both inward- and outward-open conformational states, allowing substrate transport through “alternating access” [15], and also demonstrated the presence of additional intermediate states for at least some transporters [14].

Each NBD binds an ATP molecule which is asymmetrically sandwiched by the other NBD. Therefore each nucleotide binding site (NBS) consists of conserved sequence motifs from both NBDs. This architecture was first predicted [16], then confirmed by homodimeric structures of the DNA repair protein Rad50 isolated NBD [17], the bacterial transporter NBD subunit MJ0796 [18], and shortly thereafter in a full-length transporter [13]. ABC transporter structures also illustrate how the NBDs alternate between a closed, ATP-dependent, sandwich conformation, and various open states. For example, the outward-open structure of the bacterial drug exporter Sav1866 [8] showed a fully engaged NBD dimer, whereas inward-open structures of p-glycoprotein multidrug efflux pumps revealed fully disengaged NBDs separated by 30 Å [19, 20]. Other structures display partially engaged [21] or separated NBDs [9, 22]. A common theme is that closed NBD conformations are generally associated with outward-facing states, and disengaged NBDs with inward-facing states.

While these structures reveal distinct states, models describing the whole catalytic cycle for a given transporter are now slowly emerging. Consistent with the “switch” model, in which both ATPs are hydrolyzed sequentially resulting in disengagement of the NBDs [23, 24], a state with fully disengaged NBDs has been observed In the maltose transporter [25, 26]. In contrast, in the "constant contact" model the NBDs never fully disengage, either alternating which NBS hydrolyzes ATP or preferentially hydrolyzing at one site in an asymmetric transporter [27, 28]. Both models may be represented in nature, and it was recently suggested that largely symmetric transporters, such as homodimeric MsbA, may favor a switch mechanism, while asymmetric transporters, like BmrCD, may favor a constant-contact mechanism [29].

One important model system for asymmetric ABC transporters is the vertebrate transporter associated with antigen processing (TAP) [30, 31]. TAP is in the endoplasmic reticulum (ER) membrane where it exports cytosolic peptides into ER lumen. These peptides are loaded onto MHC I molecules for scanning for foreign antigens by T-cells [30]. TAP is a heterodimer of TAP1 and TAP2, each comprising a TMD followed by an NBD. Therefore its two distinct NBSs are asymmetric, one containing consensus sequence motifs, while the second has several degenerate motifs—motifs that deviate considerably from the consensus sequences.

We previously found that unlike most isolated NBDs that function as physiological heterodimers, the rat TAP1 NBD forms ATP-hydrolyzing homodimers in vitro [32]. The isolated first NBD of the cystic fibrosis transmembrane regulator (CFTR), which also homodimerizes in vitro, is another notable exception [33]. We took advantage of the non-physiological rat TAP1 NBD homodimer as a model system to study molecular mechanisms of dimerization and ATP hydrolysis [32, 34, 35].

NBD dimerization is generally ATP-dependent and finely regulated in each ABC transporter: the TMDs modify both affinity and specificity of dimerization, and allosteric interactions with transport substrates and other regulatory factors also affect NBD dimerization. However, we still do not understand all the molecular determinants within the NBDs that can alter dimerization propensity. Here we compared the homodimerization properties of the isolated human and rat TAP1 NBDs. Despite high sequence identity, the human TAP1 NBD does not form homodimers in solution, unlike its rat counterpart. We then generated reciprocal chimeric constructs, engineering an ATP-dependent human TAP1 NBD homodimer and a monomeric rat TAP1 NBD. From these results, we pinpoint the D-helix as the structural element important in tuning the dimerization affinity of these TAP1 NBDs.

## Materials and methods

### Protein expression and purification

Plasmids based on the pET21(a) vector (Novagen) for expression of isolated WT human and rat TAP1 NBDs (residues 489–748 and 466–725 for human and rat TAP1, respectively) as C-terminally 6-His-tagged proteins were previously described [32]. All mutations were introduced using QuikChange mutagenesis (Stratagene) and confirmed by DNA sequencing (Eton Biosciences). A list of primers is provided in Table 1. The NBDs were expressed and purified as before [32]. Briefly, the proteins were expressed in *Escherichia coli* BL21(DE3), and affinity-purified on Ni-NTA resin (Qiagen), eluting with an imidazole step gradient. Fractions containing the NBD were further purified on a Superdex200 size-exclusion chromatography (SEC) column (GE Healthcare), eluted with 20 mM Tris-HCl (pH 8.0), 50 mM NaCl, 5 mM MgCl_2_, 10% glycerol, 1 mM ATP and 1 mM DTT. Relevant fractions were pooled, concentrated to 10 mg/ml, aliquoted and stored at -80°C.

**Table 1:** Primers used for mutagenesis.

Species	Mutation	Forward Primer	Reverse Primer
Human	R515H/D517N	CTACCCAAACCACCCAAATGTCTTAGTGCTACAGGGGC	CTAAGACATCTGGGCGGTTTGGGTAGGCAAAGGAGAC
	L595F/Q596R	GGTATTTGGAAGAAGTTTTCGGGAAAATATTGCCTATGGCCTGACCC	GGCAATATTTTCCCGAAAACTTCTTCCAAATACCTGTGGCTCTTGTCCC
	S623D	GTCTGGGGCCCATGATTTCATCTCTGGACTCCCTC	CCAGAGATGAAATCATGGGCCCCAGACTTTACTGCAGC
	L628F	GTTTCATCTCTGGATTCCCTCAGGGCTATGACACAG	CCCTGAGGGAATCCAGAGATGAAACTATGGGCC
	D-helix patch	CTGGATGCAGGCAACCAGTTACGGGTGCAGCGGCTCCTGTACGAAAGCC	ACAGGAGCCGCTGCACCCGTAACTGGTTGCCTGCATCCAGGGCACTG
	H702Q	CACCCAGCAACTCAGCCTGGTGGAGCAG	CCAGGCTGAGTTGCTGGGTGATGAGAAGCAC
	C-terminal patch	TGTTACCGGTCCATGGTGGAGGCTCTTGCAGCTCCTTCAGACGCGGCCGCTCATCATC	GCTGCAAGAGCCTCCACCATGGACCGGTAACACCCCCCTCTCTCCATGAGCTGCTG
	D668N	CTTATCCTGGATAATGCCACCAGTGC	CAGGGCACTGGTGGCATTATCCAGGA
	D674A	CCAGTGCCCTGGCTGCAAACAGCCAGTTACAGGTG	CTGGCTGTTTGCAGCCAGGGCACTGGTGGCATCATC
	N676G/S677N	GCCCTGGATGCAGGCAACCAGTTACAGGTGGAGCAGC	CACCTGTAACTGGTTGCCTGCATCCAGGGCACTGGTGG
	S677N	CTGGATGCAAACAACCAGTTACAGGTGGAGCAGC	ACCTGTAACTGGTTGTTTGCATCCAGGGCACTGG
Rat	D-helix patch	CCCTGGATGCTAACAGCCAGCTACAGGTCGAGCAGCTCCTGTATGAGAGCC	CATACAGGAGCTGCTCGACCTGTAGCTGGCTGTTAGCATCCAGGGCACTG
	G653N/N654S	CCCTGGATGCTAACAGCCAGCTACGGGTCCAGCG	GACCCGTAGCTGGCTGTTAGCATCCAGGGCACTGG
	D645N	CTTATCTTGGACAATGCCACCAGTGC	CAGGGCACTGGTGGCATTGTCCAAGA
	D651A	CCAGTGCCCTGGCTGCTGGCAACCAGCTACGGGTC	CTGGTTGCCAGCAGCCAGGGCACTGGTGGCATCGTC
	N654S	GATGCTGGCAGCCAGCTACGGGTCCAG	CCGTAGCTGGCTGCCAGCATCCAGGG

### Analytical SEC

For SEC experiments, 10-μl protein samples (10 mg/ml) were injected into an S200 3.2/30 column connected to an AKTA Micro (GE Healthcare). The column was pre-equilibrated with elution buffer (20 mM Tris-HCl pH 8.0, 50 mM NaCl, 5 mM MgCl_2_, 10% glycerol) including 1 mM ADP or ATP, as indicated. To minimize technical variations, all samples within a figure panel were generally run within the same sample series using the same buffers. Shown are representative traces from at least two runs. The average difference in elution volume for replicates was 0.019 mL, and the maximum was 0.030 mL.

### Analytical ultracentrifugation

Analytical ultracentrifugation (AUC) experiments were performed as described in [34]. Briefly, protein samples (33 μM) dialyzed against the sample buffer (20 mM Tris-HCl pH 8.0, 50 mM NaCl, 5 mM MgCl_2_) in the presence of the ATP or ADP. Experiments were performed in an Optima XL-I instrument equipped with interference optics (Beckman Coulter) using an An-50 Ti rotor (Beckman Coulter) and interference scans were acquired every 9 m. Buffer density, viscosity and partial specific volumes were estimated using SEDNTERP (http://sednterp.unh.edu/). Sedimentation velocity experiments were run at 40,000 rpm at 10°C for 14 h in double sector cells with sapphire windows. Data were analyzed according to continuous sedimentation coefficient distribution (c(S)) model in Sedfit [36], to determine sedimentation coefficients for each sample.

### ATP hydrolysis assay

ATP hydrolysis assays were performed using a standard colorimetric coupled assay as described in [32]. Briefly, protein samples (20 μM) were stripped of bound nucleotide by passing twice through a PD-10 column equilibrated with ATPase reaction buffer (150 mM potassium acetate, 10% glycerol, 1 mM dithiothreitol and 50 mM K-HEPES (pH 7.5)) and the A_260_/A_280_ ratio was verified to be <0.6. The ATPase reactions were conducted in 100 μL reaction buffer with 0.45 μM protein at 1.6 mM ATP. Data were collected on a SpectraMax i3 (Molecular devices) for experiments 1–3 and FlexStation 3 (Molecular Devices) for experiment 4 (see raw data files). Effective extinction coefficients at 340 nm are 1.60 or 1.87 per mM ATP for experiments run on the SpectraMax i3 and FlexStation 3, respectively. ATPase rates are presented as mean ± standard deviation from four separate experiments, each done in triplicate. Significance was evaluated in MATLAB using two-tailed Student’s t-tests testing the hypothesis at the 5% significance level, with p < 0.05 indicated in figure legends by *.

## Results and discussion

### The human TAP1 NBD does not homodimerize, unlike the rat TAP1 NBD

**Fig 1:**
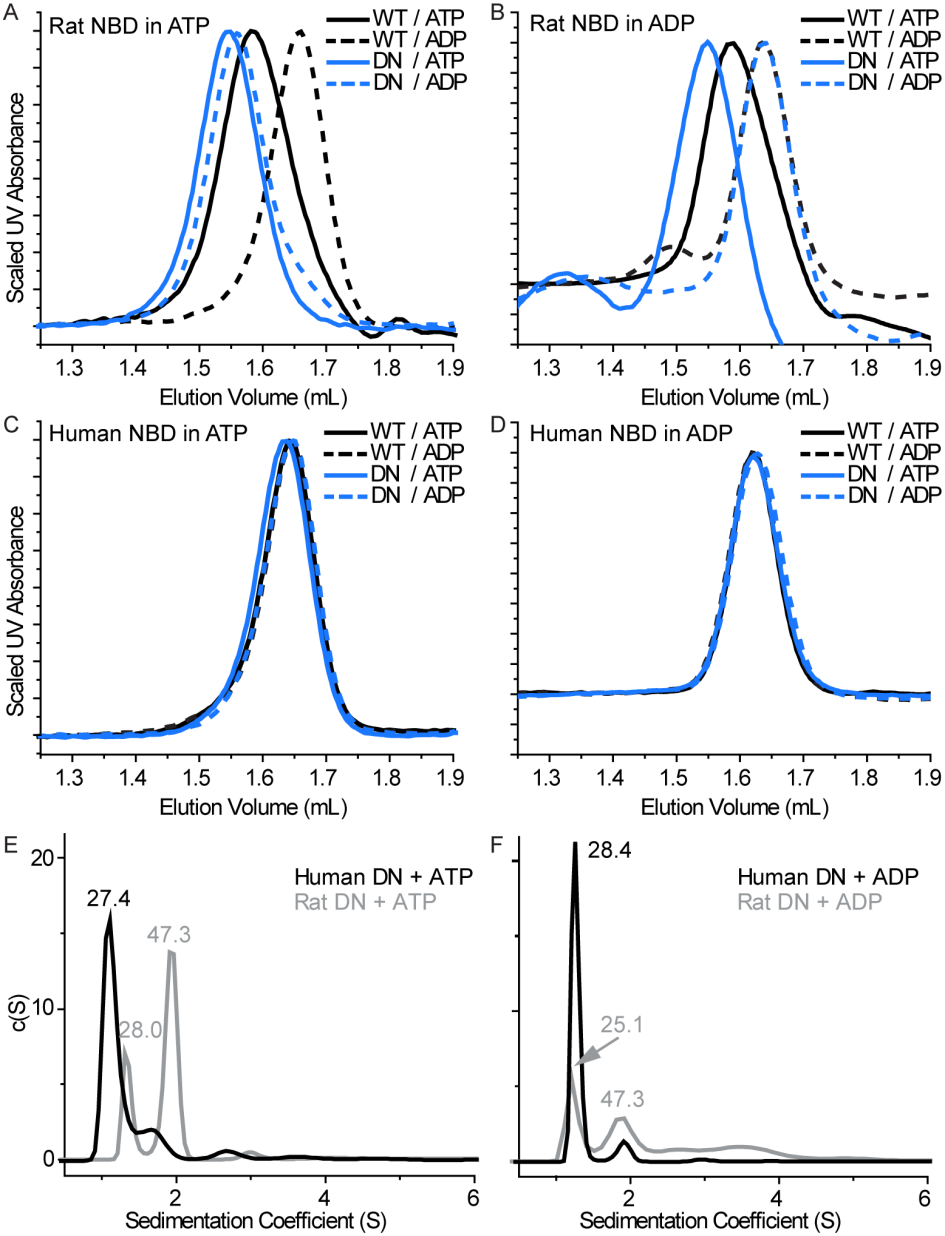
Human TAP1 NBD does not form homodimers. (**A-D**) SEC elution profiles for rat (**A** and **B**) and human (**C** and **D**) WT and Walker B D-to-N NBD variants. Panels **A** and **C** show results for proteins preloaded with ATP, while panels **B** and **D** show results for proteins preloaded with ADP. The nucleotide present in the SEC elution buffer is indicated in the legends at the top right of each panel. Traces are representatives of at least two replicates, and for at least two independent protein preparations for each NBD. (**E-F**) Sedimentation coefficient distribution from SV experiments (performed once) on the human and rat Walker B D-to-N NBD variants in the presence of ATP (**E**) and ADP (**F**). The data for rat TAP1 NBD are previously published [34] and included here for comparison.

We previously took advantage of the fact that the isolated rat TAP1 NBD forms an unphysiological homodimer to study the role of various conserved motifs in dimerization and ATP hydrolysis [32, 34, 35]. Here, we compared the dimerization properties of the rat and human TAP1 NBDs, using analytical size exclusion chromatography (SEC). Fig 1A and 1B show the behavior of the rat TAP1 NBD preloaded with ATP or ADP, respectively, and run on SEC in buffer containing either ATP or ADP. Consistent with our earlier findings [32], the wildtype (WT) rat TAP1 NBD eluted at a volume consistent with a monomer in the presence of ADP in the running buffer, and showed a leftward shift in the presence of ATP (Fig 1A). Introducing the classical Walker B mutation (D645N), which impairs ATP hydrolysis [32], led to the observation of a stable dimeric state in the presence of ATP (Fig 1A). When WT rat TAP1 NBD and the D645N variant were preloaded with ADP, both eluted as monomers in the presence of ADP (Fig 1B). In contrast in ATP-containing SEC buffer, both rat TAP1 NBD variants eluted at a larger apparent molecular weight, consistent with at least some dimerization induced by exchange of ADP for ATP (Fig 1B). Furthermore, in ADP-containing SEC buffer, the ATP-loaded D645N still showed an SEC peak largely consistent with a dimeric state (Fig 1A), indicating that ATP hydrolysis and/or nucleotide exchange is very slow in this mutant. By comparison, we conclude that the WT TAP1 NBD peak eluted in ATP-containing buffer is consistent with a mixture of dimer and monomer, as we previously confirmed by multi-angle laser light scattering [32]. Overall, these data confirm the previously observed ATP-driven NBD homodimerization of the rat TAP1 NBD [32].

We performed the same SEC experiments on both WT human TAP1 NBD and the corresponding Walker B mutant, D668N. Both human TAP1 NBD variants eluted at a volume consistent with a monomeric form under all tested conditions (Fig 1C and 1D). These results indicate that unlike rat TAP1 NBD, the isolated human TAP1 NBD does not readily show ATP-dependent homodimerization.

We performed analytical ultracentrifugation (AUC) experiments to confirm the monomeric state of the human TAP1 NBD, using the D668N hydrolysis-deficient variant to insure that the protein population was essentially all in the ATP-bound state. Our previously published sedimentation coefficient distribution from sedimentation velocity (SV) experiments for the rat TAP1 D645N NBD variant, reproduced here in Fig 1E for comparison, showed two main peaks in the presence of ATP in the sedimentation coefficient distribution, corresponding to molecular weights of 28.0 and 47.3 kDa consistent with monomeric and dimeric NBDs [34]. However the sedimentation coefficient distribution of the human TAP1 D668N NBD variant showed only one main peak with a molecular weight of 27.4 kDa corresponding to monomeric form. As expected, in the presence of ADP both rat and human NBDs appear mainly as monomers (Fig 1F). In addition, the ratio of the two most abundant species in the SV experiments, represented by the ratio of the area under the peaks assigned as monomer and dimer species, are again consistent with the rat TAP1 NBD forming an ATP-dependent homodimer, while the human TAP1 NBD was essentially monomeric in solution (Table 2). These SV results are in agreement with the SEC data, demonstrating that the isolated human TAP1 NBD did not form homodimers under the tested conditions.

**Table 2:** Data analysis for the sedimentation velocity experiments.

TAP1 NBD variant	Nucleotide	Molecular weight in kDa		2^nd^/1^st^ ratio
		1^st^ peak (monomer)	2^nd^ peak (dimer)	
Rat D645N*	ATP	28.0 (1.14)^†^	47.3 (2.97)^†^	2.60
Rat D645N*	ADP	25.1 (1.20)	47.3 (0.82)	0.68
Human D668N	ATP	27.4 (4.03)	49.5 (0.85)	0.21
Human D668N	ADP	28.4 (2.88)	52.6 (0.30)	0.10

*Data from [34] included for comparison.

^†^Numbers in parentheses are the area under the curve for the peaks in SV runs.

### Engineering a homodimerizing human TAP1 NBD

**Fig 2:**
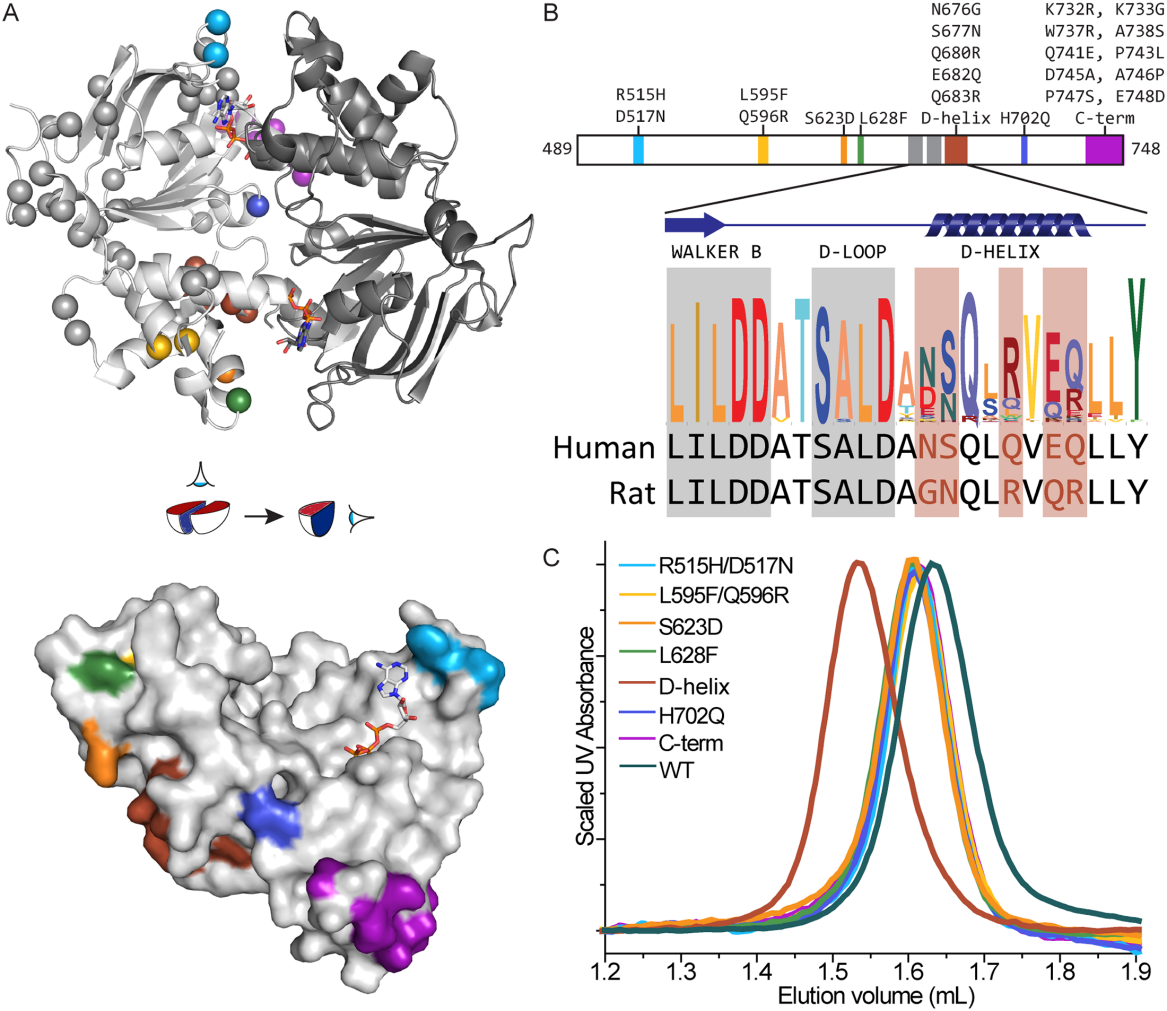
Human-to-rat substitutions in the D-helix enable human TAP1 NBD homodimerization. The seven sets of substitutions tested are shown in different colors, which are consistent across all panels. (**A**) At the top is the homodimeric rat TAP1 D645N NBD structure (PDB ID 2IXE; [32]) shown as a cartoon representation viewed from the membrane plane, with one protomer in light grey and the other in dark grey. On the left protomer, the 56 residues differing between human and rat TAP1 are indicated by Cα atom spheres. At the bottom is a surface representation of one protomer, showing the dimerizing surface, with the membrane plane at the top. The cartoon in the middle illustrates how the two views are related. The tested substitutions (colored) were focused on the dimerization interface. (**B**) Scaled linear representation of the human TAP1 NBD with the tested substitutions indicated above. The inset shows a sequence logo generated by Skylign (www.skylign.org) from 64 eukaryotic TAP1 sequences for the Walker B and D-helix region, highlighting the "D-helix" substitution set. (**C**) SEC elution profiles of protein samples preloaded with ATP and run in ATP-containing buffer show that only the D-helix variant elutes as a homodimer. Traces are representatives of two replicates from one protein preparation for each NBD.

To better understand the homodimerization of our rat TAP1 NBD model system, we took advantage of the high sequence identity between rat and human TAP1 to identify the sequence differences responsible for their differing behaviors. That is, we tested whether we could engineer a homodimerizing human TAP1 NBD by introducing one or more human-to-rat substitutions. With 76.2% sequence identity between the human and rat TAP1 NBDs, a total of 56 residues differ between the two sequences. The positions of these differences are shown on the homodimeric rat TAP1 NBD structure in Fig 2A [32]. While many differences are far from the homodimerization interface, sequence differences near the interface are good candidates to explain the differing dimerization properties of the human and rat TAP1 NBDs. We engineered seven human TAP1 NBD variants, each with one or more human-to-rat substitutions—grouping nearby residues (Fig 2A and 2B). We purified these seven chimeric human TAP1 NBD constructs and tested whether they formed ATP-dependent homodimers by analytical SEC. While six of the seven chimeric constructs behaved as monomers, the D-helix construct eluted at an apparent molecular weight consistent with a dimer (Fig 2C), This D-helix construct contains five human-to-rat substitutions in the α-helix C-terminal to the conserved D-loop: N676G, S677N, Q680R, E682Q and Q683R (Fig 2B). Thus the D-helix sequence plays an important role in mediating NBD dimerization.

### Mutation of the D-loop aspartate impairs dimerization of the human TAP1 NBD D-helix variant

**Fig 3:**
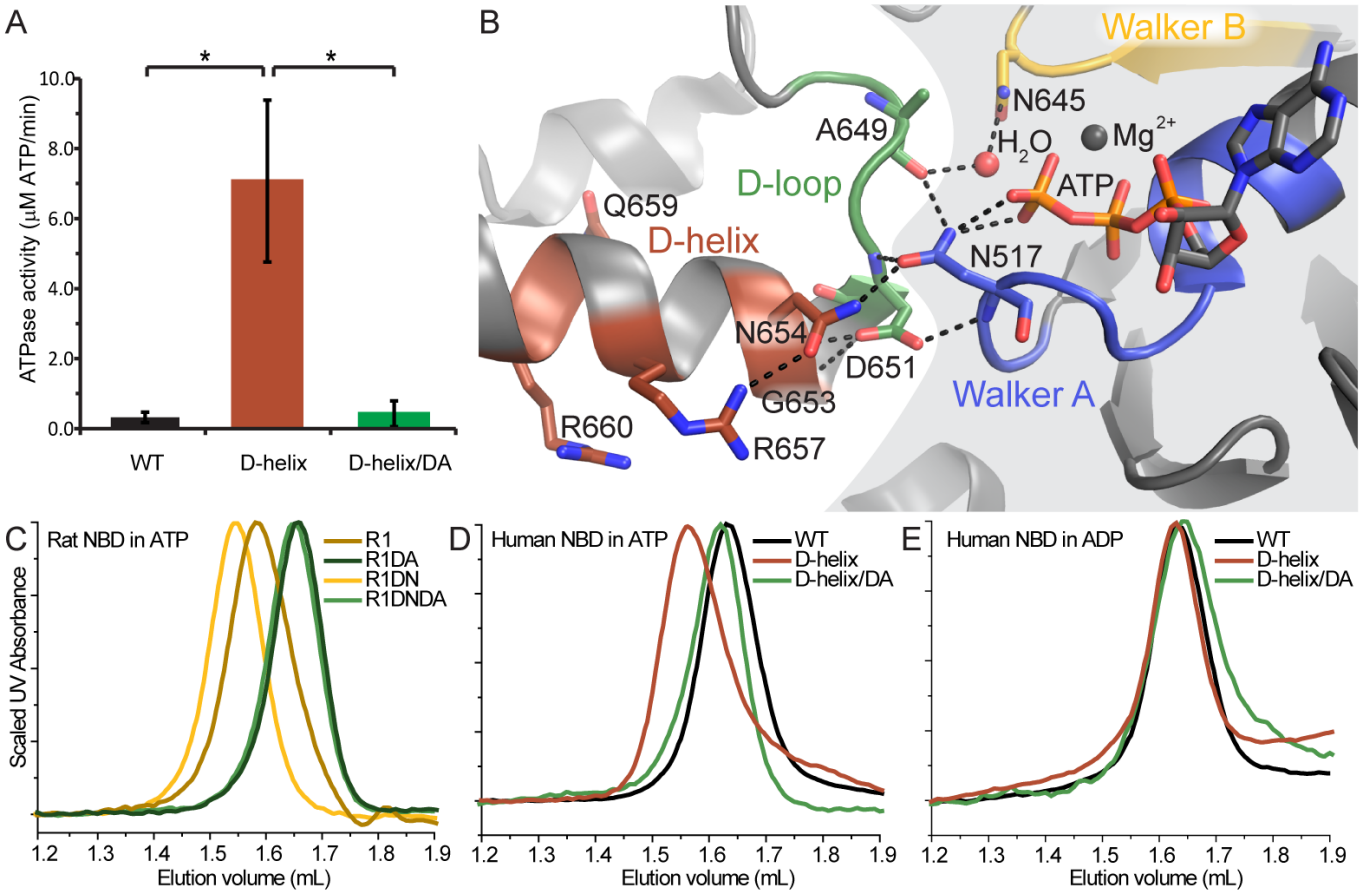
D-loop D674A mutation impairs engineered human TAP1 NBD dimerization and ATPase activity. (**A**) ATPase activity parallels the SEC dimerization data, showing higher ATPase activity for the dimerization-competent human TAP1 D-helix NBD variant compared to WT human TAP1 NBD, and significantly decreased ATPase activity when the D-loop D674A mutation is introduced in the human D-helix variant. ATPase rates are mean ± standard deviation from four separate experiments, each done in triplicate. Significance was evaluated using two-tailed Student’s t-tests, with p < 0.05 indicated by *. (**B**) The relevant part of the interface from the rat TAP1 NBD structure (PDB ID 2IXE; [32]), showing the D-loop and D-helix from one protomer on the left, and the Walker A and B motifs from the other protomer on the right, forming an interaction network around the ATP γ-phosphate. Note that the corresponding variant D-helix residues in rat/human are: G653/N676, N654/S677, R657/Q680, Q659/E682, and R660/Q683. The grey background delineates the protomer on the right. (**C-D**) SEC traces of protein samples preloaded with ATP and run in ATP-containing buffer showing that a D-to-A mutation in the D-loop impaired dimerization of the rat TAP1 NBD and its D645N variant (**C**) and the human TAP1 D-helix NBD variant (**D**), as evidenced by a right-shift of their elution volumes. (**E)** Dimerization of the human TAP1 D-helix NBD is ATP dependent as its elution was right-shifted when run in ADP-containing buffer. All SEC traces are representatives of at least two replicates from one protein preparation for each NBD. The SEC traces for the rat D645N and human D-helix variants are reproduced from Figs 1A and 2C, respectively, for comparison.

We next sought to confirm that the human TAP1 D-helix NBD variant dimerizes using the canonical ATP-sandwich interface that enables ATP hydrolysis. The D-helix variant indeed showed increased ATPase activity in comparison to the WT human TAP1 NBD (Fig 3A). We further took advantage of the distinct phenotype of the aspartate-to-alanine (D674A) mutation in the highly conserved D-loop of human TAP1 (Fig 3B; [34]). In the context of the full-length TAP transporter, D674A impairs ATPase activity, but not peptide transport, converting TAP into a nucleotide-dependent facilitator [34]. Similarly, the D-loop D651A mutation impairs dimerization and ATPase activity of the isolated rat TAP1 NBD [34]. Consistently, rat TAP1 D651A NBD behaves as a monomer on SEC (Fig 3C). When introduced into the dimer-forming human TAP1 D-helix NBD variant, the D-loop D674A mutation eluted similarly to the monomeric WT human TAP1-NBD, indicating that the mutation impaired dimerization (Fig 3D). Accordingly, the D674A mutation also significantly reduced the ATPase activity (Fig 3A). Overall, the ATPase assay results parallel the dimerization data such that only the dimer-forming D-helix variant showed significantly levels of ATPase activity. Importantly, the human TAP1 D-helix NBD elutes consistently with a monomer in SEC experiments in the presence of ADP, thus confirming that dimerization is ATP-dependent. Therefore, the results in Fig 3 demonstrate that the engineered human TAP1 D-helix NBD variant homodimerizes using the canonical ATP-sandwich interface.

### The full D-helix human-to-rat chimera is required for human TAP1 NBD dimerization

**Fig 4:**
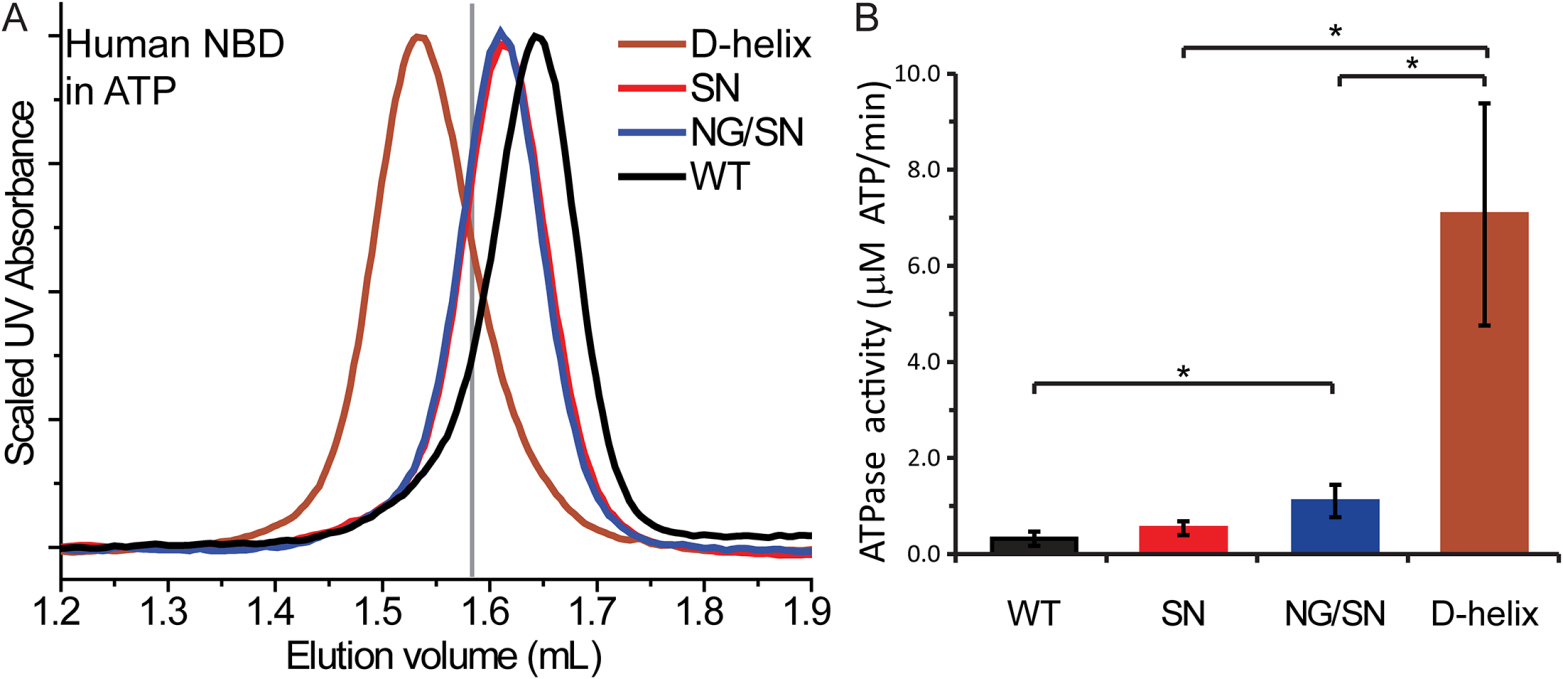
The full D-helix human-to-rat chimera is required for human TAP1 NBD dimerization. (A) The human TAP1 S677N (SN) and N676G/S677N (NG/SN) NBD variants showed an SEC elution profile most similar to WT human TAP1 NBD and therefore consistent with a monomeric state. Corresponding rat TAP1 NBD elution volume is shown with a grey line for comparison. Traces are representatives of three replicates from two protein preparation for each NBD. (B) In agreement with their lack of dimerization, the S677N (SN) and N676G/S677N (NG/SN) NBD variants showed little ATPase activity, shown as mean ± standard deviation from four separate experiments, each done in triplicate. Significance was evaluated using two-tailed Student’s t-tests, with p < 0.05 indicated by *.

The human TAP1 D-helix NBD construct contains five mutations in the α-helix that follows the D-loop. To determine whether only the mutations nearest the dimerization interface are necessary to convert the human TAP1 NBD into an ATP-dependent homodimer, we introduced single (S677N) or double (N676G/S677N) mutations into the human TAP1 NBD. We chose these residues, corresponding to G653 and N654 in the rat TAP1 NBD, because of their proximity to the dimer interface (Fig 3B). However, both the S677N and N676G/S677N variants showed SEC profiles most consistent with impaired dimerization (Fig 4A). Consistent with their impaired dimerization, both S677N and N676G/S677N show significantly lower ATPase rate than the D-helix variant (Fig 4B). Therefore, we conclude that the mutations nearest the dimer interface are not solely responsible for the dimerization and higher ATP hydrolysis rates of the human TAP1 D-helix NBD variant. Rather, some or all of the additional three mutations along the D-helix contribute to this phenotype, suggesting that the overall positioning of the D-helix has an important role in canonical dimer formation.

### Rat-to-human D-helix mutations disrupt rat TAP1 NBD homodimerization

**Fig 5:**
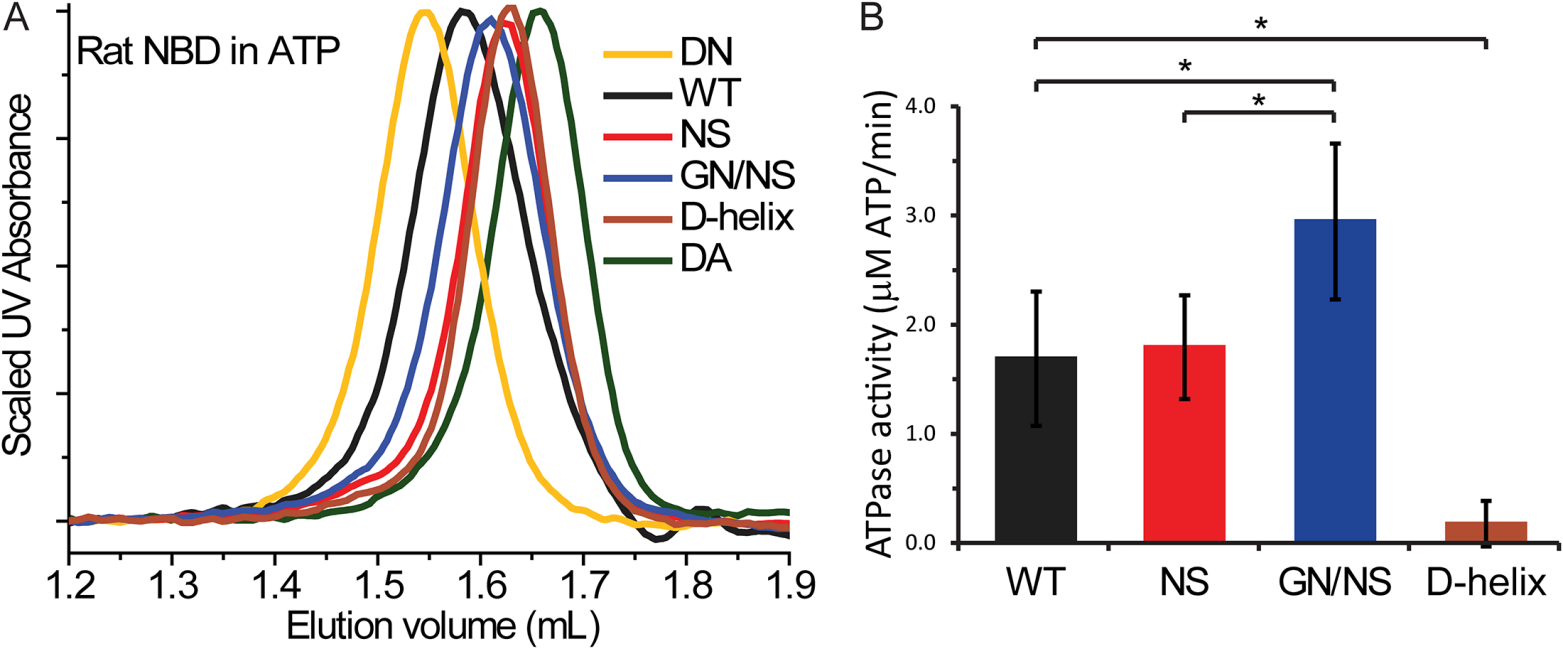
Introducing the human D-helix sequence into the rat TAP1 NBD impairs homodimerization. (**A**) SEC traces show that introducing the single N654S mutation or the five-mutation rat-to-human D-helix swap results in a right shift in the elution volume, indicating impaired dimerization, whereas the G653N/N654S double mutant elutes as an apparent mixture of monomers and dimers. Traces are representatives of two replicates from one protein preparation for each NBD. (**B**) ATPase activity measurements of the rat-to-human D-helix variants show that ATPase activity, shown as mean ± standard deviation from four separate experiments, each done in triplicate, is sensitive to the D-helix sequence. Significance was evaluated using two-tailed Student’s t-tests, with p < 0.05 indicated by *.

To further confirm that the D-helix is an important structural element in TAP1 NBD homodimerization, we sought to disrupt homodimerization by introducing rat-to-human mutations in the WT rat TAP1 NBD background. We generated three constructs, one introducing all five changes (D-helix; G653N, N654S, R657Q, Q659E and R660Q), and two introducing changes only at residues closest to the dimer interface, G653N/N654S and N654S, respectively. Analytical SEC showed that introducing all five changes (D-helix) impairs dimer formation as evidenced by a right-shift in the elution volume (Fig 5A). Consistent with this finding, the ATP hydrolysis rates for this construct is significantly lower than the WT rate (Fig 5B). The N654S and G653N/N654S variants showed mixed phenotypes, with statistically similar ATPase rates to WT and significantly higher ATPase rates than WT, respectively, but elution volumes consistent with less stable dimerization than the D-helix variant (Fig 5A). Taken together, these results suggest that the D-helix has an important role in NBD dimerization, and that the dimerization equilibrium and ATPase activity are both highly sensitive to the D-helix sequence and structure.

## Conclusion

Using human-rat chimeric constructs, we identified the D-helix, which follows the highly conserved D-loop motif, as an important determinant of ABC-type NBD dimerization for the TAP1 NBD. We showed that a set of five mutations converting the D-helix to its rat sequence enables homodimerization of the isolated human TAP1 NBD, and as a result, enables ATP hydrolysis. Conversely, the corresponding five mutations to convert the D-helix to its human sequence in the isolated rat TAP1 NBD impaired its ability to homodimerize, and concomitantly, its ATPase activity. Additional mutational analysis of the D-helix region also highlights that NBD dimerization is highly sensitive its precise sequence, and therefore structure.

The rat TAP1 NBD homodimer structure shows the canonical ATP-sandwich NBD dimer interface [32]. N654 from the D-helix directly interacts with N517 in the Walker A motif of the second protomer (Fig 3B). The Walker A N517 also interacts with the γ-phosphate of the bound ATP, aligning it for hydrolysis. Therefore, the structural information on ABC-type NBDs is consistent with the D-helix playing a critical role in dimer formation and ATP hydrolysis.

Our results provide additional context for interpretation of experiments on isolated ABC-type NBDs, including previous work using the homodimerizing rat TAP1 NBD [32, 34, 35]. Accumulated structural and biochemical data on the ABC-transporter family suggest that the interaction affinity of the two NBDs within a transporter should be finely tuned to function within its physiological context, and our new data suggest that the D-helix can play an important role in tuning this affinity. It is possible that in some transporters, the position of the D-helix is allosterically modulated. Indeed, there is structural evidence for important roles of the D-loop in NBD-NBD contacts in different nucleotide states [37, 38], and the D-helix shows evidence of conformational variability in maltose transporter structures [38] and Sav1866 molecular dynamics simulations [39]. Future biochemical, structural, and bioinformatics analyses on the ABC transporter family will continue to shed light on the sequence determinants that enable the conformational cycling associated with substrate transport by ABC transporters.

## Supporting information

S1 File Text file containing the calculated ATP hydrolysis rates illustrated in Figs 3, 4 and 5. (TXT)

S2 File Text file containing the raw ATP hydrolysis data for the human TAP1 NBD variants illustrated in Figs 3 and 4. (TXT)

S3 File Text file containing the raw ATP hydrolysis data for the rat TAP1 NBD variants illustrated in Fig 5. (TXT)

S4 File Text file containing the raw SEC data for the human TAP1 NBD variants run in the presence of ATP. (TXT)

S5 File Text file containing the raw SEC data for the human TAP1 NBD variants run in the presence of ADP. (TXT)
